# Editorial: Insights in biofilms: 2021

**DOI:** 10.3389/fcimb.2022.1093692

**Published:** 2022-11-23

**Authors:** Brendan F. Gilmore, Diane McDougald, Christophe Beloin

**Affiliations:** ^1^ Queen’s University Belfast, School of Pharmacy, Biofilm Research Group, Belfast, United Kingdom; ^2^ Australian Institute for Microbiology & Infection, University of Technology Sydney, Sydney, NSW, Australia; ^3^ Institut Pasteur, Université de Paris Cité, UMR CNRS 6047, Genetics of Biofilms Laboratory, Paris, France

**Keywords:** aggregates, antibiofilm, models, polymicrobial, physiology

Despite over four decades of research into microbial biofilms, the clinical management of biofilm-mediated infections remains problematic. Environmental biofilms contribute to massive economic losses, estimated in the region of $5 trillion USD annually, and are potential reservoirs for pathogens and antimicrobial resistance genes (ARGs) (Cámara et al., 2022; Highmore et al., 2022). The aim of this special Research Topic is to bring together recent insights into influences of the host environment, aggregates in *in vivo* infections, microbial biofilm physiology and emerging approaches for biofilm control.

In a wide-ranging and comprehensive review, Hall-Stoodley and McCoy examine the role of the host environment, specifically mucosal epithelial microenvironments, on biofilm formation in the respiratory tract and the contribution of airway pathophysiology and altered barrier and clearance mechanisms (often the result of microbial colonisation). This review focussed on bacterial aggregates, defined as cohesive groups of microbial cells surrounded by extracellular polymeric substances (EPS) which exhibit a biofilm-like phenotype (Sauer et al., 2022). These aggregates may be suspended within the mucus hydrogel layer of the airways, free floating or directly adhered to epithelial cells. In either case, the higher order multicellular organisation of bacteria within these aggregates and diversity of bacterial phenotypes allow aggregated microorganisms to tolerate stressful conditions encountered within the host. The role of microenvironmental conditions of, for example, the CF lung in promotion of this aggregative phenotype is described, and insights into more accurate modelling of the human airway microenvironment are discussed, to facilitate and inform ongoing research into improving the management of biofilms in respiratory tract infections.

The perspective by Oliveira et al. reviews the role of iron metabolism in *S. epidermidis* pathogenesis and biofilm formation, and considers the potential of targeting iron-acquisition processes for development of novel anti-infective strategies. Building on the themes discussed in the review by Hall-Stoodley and McCoy, biofilm formation is influenced by the host environment and represents an effective strategy to withstand the harsh environment encountered during colonisation, which includes “nutritional immunity”. Understanding the microenvironment of infectious diseases is essential to identify novel strategies to fight pathogens (Bjarnsholt et al., 2022). In the human body, free iron (as the ferric ion, Fe^3+^) is present in extremely low concentrations (estimated to be as low as 10^-24^ M), below those normally required to sustain bacterial growth and proliferation (c. 10^-6^ M). Pathogens must therefore scavenge iron and other essential metals in order to successfully colonise the host. Previous RNAseq studies have shown that iron-metabolism genes are most upregulated when *S. epidermidis* is exposed to human blood, which suggests a role for iron acquisition in biofilm formation and survival within the human host (França et al., 2014).

Despite this, there remains a paucity of studies in the literature dealing with iron acquisition mechanisms in *S. epidermidis*. Increasing evidence points to iron exerting a modulatory effect on *S. epidermidis* biofilm formation, but the putative regulatory role of iron in *S. epidermidis* biofilm is not well understood. In addition, previous studies show that inactivation of iron acquisition systems leads to measurable loss in virulence, and catecholamine inotropic drugs, frequently administered to patients in intensive care units, promote biofilm formation through sequestration of iron from transferrin. The authors review the potential for a ‘trojan horse’ strategy which exploits iron uptake systems, that may be used to improve delivery of antibiofilm compounds and conventional antibiotics to staphylococcal cells and biofilms, through conjugation of compounds to siderophores or exploitation of heme transport systems. Given the emerging evidence for the central role of iron acquisition in *S. epidermidis* biofilm formation and virulence, exploitation of these processes could deliver much needed innovation in development of new therapeutic agents and strategies to control this important pathogen.

In addition to exploit iron uptake systems identification of novel agents to fight biofilms is another option to help clinicians to tackle biofilm-related infections. The management of endodontic infections is complicated by formation of microbial biofilms, which are implicated in root canal treatment failure, persistent infection and tolerance to bactericidal agents employed in their clinical management. Nassar et al. describe the antimicrobial activity of phytic acid (also inositol hexaphosphate, IP6), as a potential emerging agent for biofilm control endodontic procedures. Phytic acid, a natural organic acid, represents an attractive alternative to EDTA in endodontic irrigants. Having a high negative charge density rendering it capable of chelation of cations, phytic acid is able to remove the smear layer (a layer of organic material deposited on the canal walls following endodontic surgery, comprising pulp tissue, dentine, odontoblastic processes and often microorganisms) and is biocompatible. The authors demonstrate that IP6 exhibited broad spectrum antimicrobial activity, bactericidal activity confirmed against *E. faecalis* after a contact time of 5 mins (a time consistent with current clinical practice). The activity of IP6 was compared against EDTA and sodium hypochlorite, the main irrigants for root canal treatment in current clinical use. Phytic acid exhibited activity against both planktonic microorganisms and biofilms, at concentrations of 0.5 – 2%. Concentrations of 2% exerted a bactericidal effect on *E. faecalis* after 5 mins, and 5% IP6 was bactericidal after 30 sec contact time. Compared to EDTA, IP6 exhibits a broader antimicrobial spectrum at significantly lower concentrations. EDTA also lacked activity against biofilms, including *E. faecalis* and *C. albicans*. Phytic acid could be a useful alternative to EDTA in the control of endodontic biofilms, associated with recurrent, difficult to treat root canal infections in patients.

The development and application of appropriate *in vitro* and *in vivo* biofilm models are central not only to understanding biofilm physiology and accounting for role of the host microenvironment of biofilm formation, but are essential for advancement of accurate diagnostic and antimicrobial/antibiofilm screening and discovery platforms (Lebeaux et al., 2013; Brackman and Coenye, 2016; Malone et al., 2017). Wu et al. describe the development of a PF (Poly(ethylene glycol) diacrylate, PEGDA, linked to a Fibrinogen backbone) hydrogel biofilm implant model for the study of *Pseudomonas aeruginosa* biofilms *in vitro* and *in vivo*. *P. aeruginosa* embedded within the PF hydrogel matrix were shown to develop typical biofilms *in vitro* and in an *in vivo* mouse peritoneal cavity model, and exhibited antibiotic tolerance (>256x MIC) and were protected from the host immune response, despite excessive infiltration of the PF hydrogel by polymorphonuclear leukocytes (PMNs) when placed *in vitro*. This system represents a facile, reproducible biofilm model which displays features of real biofilm infections, with a potentially diverse range of future applications in studying biofilm development, chronicity, antimicrobial tolerance, host immune tolerance and efficacy of new therapeutic agents and approaches.

One way to increase the relevance of fundamental studies is to integrate the polymicrobial nature of numerous infections that can strongly impact the outcome of treatments (Orazi and O’Toole, 2019; Zhang et al., 2022). The opportunistic pathogen *Candida albicans* participates in formation of polymicrobial biofilms with a range of bacteria, in a number of clinical scenarios, often leading to consequences which determine the outcome of co-infections (Ponde et al., 2021). The review article by Pohl highlights the major advances in the study of polymicrobial biofilms formed by *C. albicans* and bacteria over the period 2016-2021. Among these, are the development of new models to study *C. albicans* polymicrobial biofilms and discussion of the challenges in complex model design to facilitate study of polymicrobial, cross-kingdom interactions. The role of these complex interactions is central to our understanding their impact on pathogenesis, host immune response and antimicrobial tolerance and resistance. Polymicrobial biofilm models have also supported significant research efforts in the screening of natural products, quorum sensing (QS) molecules (including the *C. albicans* QS molecule, farnesol) and QS-regulated molecules such as phenazine, alongside antibiotics, antifungals and antibiotic-antifungal synergy screens, for polymicrobial biofilm control. Whilst significant challenges exist in the study of these complex multi-species biofilm communities, tools such as metatranscriptomics and metagenomics are likely to facilitate more in-depth studies of the complex cellular and molecular interactions occurring within biofilms formed by *C. albicans* and bacteria, and derive novel insights for their successful clinical management.

## Author contributions

BG wrote the first draft. CB and DM provided critical comments and editorial suggestions for revisions. All the authors agreed on the submitted version.

## Acknowledgments

We gratefully acknowledge the authors and reviewers who have participated in this Research Topic.

## Conflict of interest

The authors declare that the research was conducted in the absence of any commercial or financial relationships that could be construed as a potential conflict of interest.

## Publisher’s note

All claims expressed in this article are solely those of the authors and do not necessarily represent those of their affiliated organizations, or those of the publisher, the editors and the reviewers. Any product that may be evaluated in this article, or claim that may be made by its manufacturer, is not guaranteed or endorsed by the publisher.
